# GDNF‐RET signaling drives pulmonary neuroendocrine cell hyperplasia and allergic airway inflammation

**DOI:** 10.1002/1873-3468.70341

**Published:** 2026-04-12

**Authors:** Tasuku Kawano, Erina Ike, Suguru Okada, Tomoko Takahashi

**Affiliations:** ^1^ Division of Pathophysiology, Department of Pharmaceutical Sciences, Faculty of Pharmaceutical Sciences Tohoku Medical and Pharmaceutical University Sendai Japan

**Keywords:** asthma, GDNF, GFRα1, group 2 innate lymphoid cells (ILC2), neuroimmune interaction, pralsetinib, RET

## Abstract

Pulmonary neuroendocrine cell (PNEC) hyperplasia often occurs in lung diseases, including allergic asthma. We previously reported that PNEC‐derived calcitonin gene‐related peptide (CGRP) likely stimulates group 2 innate lymphoid cells (ILC2), exacerbating asthma phenotypes in a mouse model. Here, we investigate the role of glial cell‐line derived neurotrophic factor (GDNF) and rearranged during transfection (RET) signaling in PNEC hyperplasia and its therapeutic potential in asthma. PNECs expressed GDNF receptors, which were activated primarily by infiltrating inflammatory cells. Application of a RET‐specific inhibitor suppressed ILC2 levels, PNEC hyperplasia and airway allergic responses. We suggest that GDNF‐RET signaling promotes PNEC hyperplasia and that the PNEC‐CGRP‐ILC2 axis is closely associated with the development of allergic asthma, presenting a possible new treatment strategy.

**Impact statement**

Our study is the first to indicate the possibility of controlling pulmonary neuroendocrine cell (PNEC) hyperplasia and acute allergic airway inflammation through RET signaling, which could lead to elucidating the mechanism underlying the PNEC hyperplasia‐immune relationship in asthma. We propose that targeting this could be a new treatment strategy.

## Graphical abstract

A neuroimmune interaction centered on the PNEC‐ILC2 axis in an asthma model. PNEC‐derived CGRP activates nearby ILC2s, which then release cytokines, including IL‐5 and IL‐13. These cytokines promote eosinophil and macrophage infiltration. Subsequently, GDNF produced by these cells induces PNEC hyperplasia via the GDNF receptors, RET and GFRα1. Targeting this pathway could provide a new approach for treating allergic asthma.

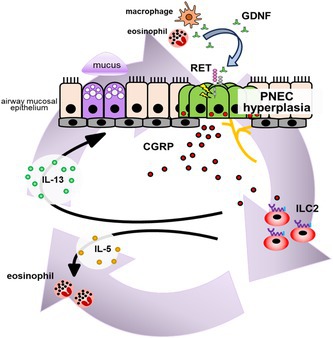

## Abbreviations

**CGRP**, calcitonin gene‐related peptide

**GDNF**, glial cell‐line derived neurotrophic factor

**GFRα1**, GDNF family receptor alpha 1

**ILC2**, group 2 innate lymphoid cells

**OVA**, ovalbumin

**PNEC**, pulmonary neuroendocrine cell

**RET**, rearranged during transfection

**Th2**, T helper 2

Allergic asthma is caused by chronic airway inflammation triggered by repeated exposure to allergens such as pollen, dust mites, and mold. Its mechanism involves increased production of allergen‐specific T helper 2 (Th2) cytokines, such as interleukin (IL)‐4, IL‐5, and IL‐13, as well as eosinophil infiltration into the airways and excessive secretion of mucus [1, 2]. Although it is well‐established that Th2 immune cells play a central role in the development and progression of allergic asthma, recent research has highlighted the importance of group 2 innate lymphoid cells (ILC2) and the interaction between the immune and nervous systems as key regulatory factors under these conditions [3, 4].

Pulmonary neuroendocrine cells (PNECs) are a distinctive type of airway epithelial cell that exhibit both neuronal and endocrine features, comprising approximately 0.5% of all airway epithelial cells [5]. PNECs exist either as solitary cells scattered throughout the airway epithelium or as innervated clusters of five to seven cells, called neuroepithelial bodies (NEBs) [6, 7]. These PNEC clusters are primarily located at bifurcation points and junctions between alveoli and terminal bronchioles, where they readily detect external stimuli, such as hypoxia, hypercapnia, mechanical stretch, and allergens [8, 9]. PNECs contain various neuropeptides and neurotransmitters, including calcitonin gene‐related peptide (CGRP), gamma‐aminobutyric acid (GABA), and serotonin, stored in dense core vesicles [10]. In response to environmental stimuli, PNECs release potent bioactive substances in a paracrine and/or endocrine manner and are believed to play a role in maintaining lung homeostasis.

PNEC hyperplasia is commonly seen in human lung diseases, including allergic asthma, chronic obstructive pulmonary disease, and cystic fibrosis [11, 12]. Additionally, congenital disorders, such as diffuse idiopathic pulmonary neuroendocrine cell hyperplasia (DIPNECH), neuroendocrine cell hyperplasia of infancy (NEHI), bronchopulmonary dysplasia, and congenital diaphragmatic hernia, are closely associated with PNEC hyperplasia [13]. Specifically, in allergic asthma, we, as well as other researchers, have recently shown that CGRP derived from PNECs activates nearby ILC2s and influences downstream immune responses, suggesting that PNEC hyperplasia could significantly contribute to the development of asthma pathogenesis and, therefore, serve as an effective target for treating asthma phenotypes [11, 14]. However, to our knowledge, the triggers of PNEC hyperplasia and their controls under these conditions remain unclear.

To address this, we focused on the glial cell line‐derived neurotrophic factor (GDNF)‐RET signaling pathway. RET encodes a receptor tyrosine kinase, which was first identified as a novel oncogene [15]. Gain‐of‐function mutations or chimeric RET have been implicated in the development of various human tumors, including multiple endocrine neoplasia types 2A and 2B, pheochromocytoma, parathyroid hyperplasia, and medullary thyroid carcinoma [16]. During embryogenesis, RET is expressed in neuronal organs and neural crest‐derived cells, some of which give rise to neuroendocrine cells, serving as a marker for both neural and neuroendocrine lineages [17]. In lung development, RET is expressed in neonatal PNECs and vagal nerves [18]. The corresponding ligands for RET belong to the GDNF family. GDNF ligands (GDNF, NRTN, ARTN, PSPN, and GDF15) primarily bind to their specific co‐receptors (GFR1 to GFR4 and GFRAL, respectively), which then trigger RET autophosphorylation [19].

In this study, we investigated the mechanism underlying PNEC hyperplasia by exploring the possible involvement of GDNF‐RET signaling and evaluating the therapeutic effects of suppressing RET activation on PNEC hyperplasia and asthma phenotypes.

## Materials and methods

### Mice

Specific pathogen‐free male C57BL/6N mice were purchased from Japan SLC Inc. and CLEA, Japan. GAD67‐green fluorescent protein (GFP) knock‐in mice (ICR.Cg‐Gad1) were provided by RIKEN BRC [20] and were backcrossed for at least eight generations with C57BL/6 strains. All animal experiments in this manuscript were carried out in accordance with both the ARRIVE guideline for reporting *in vivo* experiments and the guidelines approved by the Committee of Animal Experiments at the Tohoku Medical and Pharmaceutical University (Approval Numbers: A23015, A24013, and A25031). All experimental animals were bred in a 12 h light–dark cycle environment with free access to food and water except during the experimental period. To minimize pain and suffering, all procedures were performed under appropriate anesthesia and analgesia. Animals were euthanized when necessary by CO_2_ inhalation followed by an overdose of anesthetic.

### Asthma mouse model

An asthma mouse model was established as previously described [14]. Briefly, six‐ to seven‐week‐old mice were sensitized by intraperitoneal injection of chicken ovalbumin (OVA; Grade V; Sigma‐Aldrich, St Louis, MO, USA; 8 μg per mouse) adsorbed onto aluminum hydroxide (FUJIFILM Wako Pure Chemical Corporation, Osaka, Japan; 4 mg per mouse) on Days 0 and 5. On Days 17 and 24, all mice were challenged with aerosolized OVA (5 mg·mL^−1^) to induce an asthma‐like phenotype. Pralsetinib (Selleck Bio., Houston, TX, USA) was dissolved in DMSO (Nacalai Tesque, Kyoto, Japan) and diluted in sterile corn oil (Nacalai Tesque). The resulting solution was administered orally by gavage at 200 μL per mouse per dose (10 mg·kg^−1^; 5% DMSO and 95% corn oil). Pralsetinib was administered once‐daily from Day 18 for nine consecutive days. A vehicle control group received the corresponding solvent. Bronchoalveolar lavage (BAL) was performed [14]. Briefly, the lungs were lavaged twice by injecting 0.25 mL of cold phosphate‐buffered saline (PBS), and the recovered PBS was pooled. BALF samples were processed to count the total cell counts and cell differentials. In some experiments, lungs were harvested from a subset of mice, and homogenates were prepared for RNA and protein extraction. RNA and proteins were prepared for RT–PCR and ELISAs, respectively. Schematics of the experimental protocol are shown in Figs 2A and 4A.

### Preparation of lung cell suspensions

Lungs from C57BL/6N wild‐type and/or GAD67‐GFP knock‐in mice were excised, minced, and enzymatically digested using the enzyme mixture supplied in the Multi Tissue Dissociation Kit 1 (Miltenyi Biotec, Bergisch Gladbach, Germany). Dissociation was performed using a gentleMACS Dissociator (Miltenyi Biotec) with the 37C_n_LUNG program. The digested material was filtered through 70‐μm cell strainers, washed with phosphate‐buffered saline (PBS), and resuspended in PBS supplemented with 5 mm EDTA and 1% bovine serum albumin (PEB) buffer. The resulting lung cell suspensions were used to isolate the indicated cell populations by magnetic separation and flow cytometric sorting.

### Isolation of CD45 ^+^, CD146 ^+^, and CD31 ^+^ cells

CD45^+^ hematopoietic cells were first isolated from lung cell suspensions using CD45 MicroBeads (Miltenyi Biotec) and an autoMACS Pro Separator (Miltenyi Biotec) with the Possel program. The CD45‐depleted fraction was subsequently incubated with biotin‐conjugated anti‐CD146 antibody (BioLegend, San Diego, CA, USA), followed by Streptavidin MicroBeads (Miltenyi Biotec). CD146^+^ cells were collected using the Possel program and used as smooth muscle/perivascular cells as previously described [21]. Endothelial cells were then isolated from the remaining fraction using CD31 MicroBeads (Miltenyi Biotec) and the Possel program.

### Isolation of pulmonary neuroendocrine cells (PNECs)

To enrich epithelial cells, lung cell suspensions were incubated with PE/Cy7‐conjugated anti‐CD326 (EpCAM) antibody (BioLegend) along with CD31 and CD45 MicroBeads (Miltenyi Biotec). CD31^+^ and CD45^+^ cells were then removed using the *Depletes* program on the autoMACS Pro Separator.

The remaining cells were subsequently incubated with anti‐PE MicroBeads UltraPure (Miltenyi Biotec), and EpCAM^+^ epithelial cells were enriched using the Posseld2 program. PNECs were identified using GAD67‐GFP knock‐in mice, in which neuroendocrine cells express GFP. After staining with 7‐AAD to exclude dead cells, EpCAM^+^ GFP^+^ cells were sorted using FACSAria Fusion with FACSDiva (v9.0.1) (BD Biosciences, Franklin Lakes, NJ, USA).

### Isolation of ILC2s and Th2 cells

For isolating lymphocyte populations, lung cell suspensions were stained with biotinylated lineage markers (CD11b, CD11c, NK‐1.1, F4/80, Gr‐1, B220, CD19, and TER‐119) and incubated with Streptavidin MicroBeads (Miltenyi Biotec). Lineage‐positive cells were then depleted using the Depletes program on an autoMACS Pro Separator. The remaining cells were stained with antibodies against CD3ε, CD4, CD25, CD45.2, T1/ST2, TCRβ, Thy1.2, CD127, and KLRG1. ILC2s were identified and isolated as Lin^−^ CD3ε^−^ CD4^−^ TCRβ^−^ Thy1.2^+^ CD127^+^ KLRG1^+^ cells as previously described [14], while Th2 cells were defined as CD3ε^+^ CD4^+^ TCRβ^+^ T1/ST2^+^ cells. Cell sorting was performed using a FACSAria Fusion with FACSDiva. The gating strategies are shown in Fig. S1, and the antibodies used for flow cytometry are listed in Table S1.

### Reverse transcription‐polymerase chain reaction (RT‐PCR)

Total RNA extraction and RT‐PCR were performed as described previously [22]. Briefly, RT‐qPCR cycling conditions were as follows: an initial denaturation step at 95 °C for 10 min, followed by 40 cycles of denaturation (95 °C for 10 s) and annealing/extension (60 °C for 30 s). Fold changes in gene expression were determined using the standard curve method. The primers used for RT‐PCR are listed in Table S2.

### Periodic acid‐Schiff (PAS) staining

PAS staining was performed as previously described [14]. PAS‐stained sections were observed under a light microscope (CX43; EVIDENT, Tokyo, Japan).

### Immunohistochemistry

Immunohistochemistry was performed as previously described [14]. Primary antibodies used for immunohistochemistry are listed in Table S3. For selected paraffin‐embedded tissue sections, tyramide signal amplification (TSA)–based immunofluorescence staining was performed. Sections (6 μm thick) were deparaffinized in xylene and rehydrated in decreasing ethanol series. Antigen retrieval was performed by heating the sections in citrate buffer (pH 6.0). Endogenous peroxidase activity was quenched by incubation with hydrogen peroxide, followed by blocking of nonspecific binding using Blocking One (Nacalai Tesque). Sections were incubated with primary antibodies raised in rabbit, followed by horseradish peroxidase (HRP)–conjugated anti‐rabbit IgG secondary antibodies. Immunoreactivity was visualized using a tyramide signal amplification (TSA) system with tyramide‐Cy3 and tyramide‐Cy5 (AAT Bioquest, Inc., Pleasanton, CA, USA). For sequential multiplex staining, bound antibodies were removed by heating the sections in citrate buffer (pH 6.0), and cycles of primary antibody incubation, HRP‐conjugated secondary antibody incubation, and tyramide fluorophore labeling were repeated. Nuclei were counterstained with DAPI. All immunofluorescence images were captured using the ZEISS LSM 900 confocal laser scanning microscope.

### Enzyme‐linked immunosorbent assay (ELISA)

ELISA was performed as described previously [14]. Protein concentrations in lung homogenates, plasma, and cell culture supernatants were measured using specific ELISA kits for GDNF (Invitrogen, Carlsbad, CA, USA), as well as for IL‐5 and IL‐13 (Thermo Fisher Scientific, Waltham, MA, USA). All procedures were performed according to the manufacturer's instructions. ELISA for OVA‐IgE was performed as previously described [22, 23].

### Assessment of GDNF‐producing cells by flow cytometry

Lung cell suspensions were processed as described above. Live/dead discrimination was performed using Zombie Aqua viability dye. Cells were blocked with anti‐mouse CD16/32 (Fc) antibody and anti‐mouse IgG Fab fragment for 10 min at 4 °C. Surface staining was performed with CD11c and Siglec‐F (biotin) antibodies. After washing, cells were fixed and permeabilized using the Intracellular Fixation & Permeabilization Buffer Set (Invitrogen), and intracellular GDNF (Santa Cruz Biotechnology, 1:50) was stained at 4 °C for 60 min. Secondary detection was performed with Alexa Fluor 488‐conjugated anti‐mouse IgG for GDNF and Alexa Fluor 555‐conjugated Streptavidin for Siglec‐F. Cells were filtered through a 70‐μm strainer and analyzed on the FACSAria Fusion with FACSDiva. Eosinophils were identified as Siglec‐F^+^ and CD11c^−^ cells, while macrophages were identified as Siglec‐F^+^ and CD11c^+^ cells. The gating strategy is shown in Fig. 3A.

### Quantification of inflammatory cells in the bronchoalveolar lavage fluid (BALF)

BALF was collected 3 days after the final OVA challenge (day 27), following a previously described method [24].

### Protocol for treatment with pralsetinib

Mice with asthma were treated with either pralsetinib (10 mg·kg^−1^, BMS‐927711; Selleck Chemicals, Houston, TX, USA) or a vehicle of 5% dimethylsulfoxide and 95% corn oil via an oral sonde (Natsume Seisakusho, Tokyo, Japan) once‐daily between Days 18 and 27. The administered volume was 200 μL, and the vehicle‐treated group received an equivalent volume. The administration schedules are summarized in Fig. 4A. The dose administered was determined based on a previous study [25].

### Statistical analyses

Data are expressed as the mean ± standard deviation (SD) from multiple independent experiments (indicated by *n* values). Significant differences were determined using an unpaired *t*‐test for normal distribution and either the Mann–Whitney *U* test or one‐way analysis of variance (ANOVA) and Tukey's *post hoc* test for all other cases. Statistical analyses were performed using Prism 6 (GraphPad Software, San Diego, CA, USA). Statistical significance was set at *p* < 0.05.

## Results

### Expression of RET and GFRα1 in PNECs

**Fig. 1:**
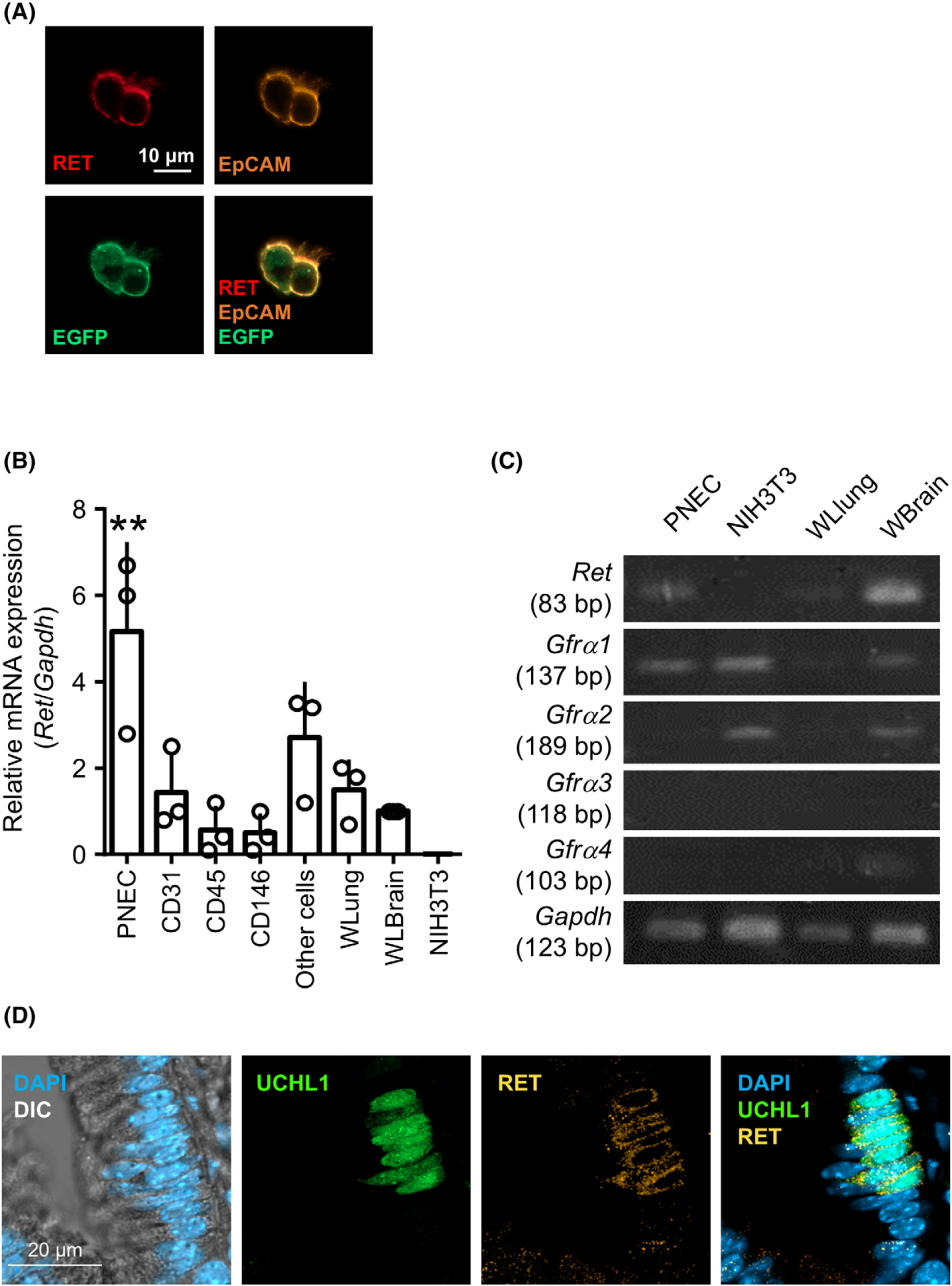
RET and GFRα1 expression in PNECs. (A) RET expression in isolated PNECs. Immunocytochemistry for RET and EpCAM in isolated PNECs. EGFP expression under the *gad67* promoter is used to isolate and identify PNECs. Scale bar, 10 μm. (B) RT‐qPCR for *Ret*. RNA obtained from each lung fraction (presented as PNEC, CD31, CD45, CD146, Other cells), whole lung (WLung), whole brain (WBrain), or NIH3T3 cell line was analyzed for RT‐qPCR. The data are shown as relative expression levels, normalized to *GAPDH*, with value for whole brain set to 1. They were analyzed using one‐way ANOVA and Tukey's *post hoc* test (*n* = 3, ** *p* < 0.01. Error bars represent mean ± SD). (C) RT‐PCR analysis of *Ret* and *Gfrα* family. mRNA obtained from isolated PNECs, NIH3T3 cell line, whole lung, and whole brain in mice. (D) Representative images of immunohistochemistry for RET in naïve lung sections. To identify PNECs, an anti‐UCHL1 antibody was used. Scale bar, 20 μm.

RET, a receptor tyrosine kinase, is primarily expressed in neuro‐ and neuroendocrine lineage cells and has been reported in neonatal PNECs [18]. Therefore, we investigated whether RET was expressed in the PNECs of adult naïve mice. Immunocytochemistry revealed that isolated PNECs expressed RET (Fig. 1A). To obtain detailed RET expression data in different lung cell populations, we classified the total lung cells into specific cell types using FACS. These included the vascular endothelial fraction (CD31‐positive), blood cell fraction (CD45‐positive), smooth muscle fraction (CD146‐positive), other cell fraction not listed above, and PNEC fraction (CD45‐negative, CD31‐negative, EpCAM‐positive, GFP‐positive), as shown in Fig. 1B. Each fraction was analyzed by RT‐qPCR. *RET* was most prominently expressed in PNECs among the lung cell populations obtained.

To transduce signals downstream of RET within cells, the RET ligand needs to bind to the cofactor GFRα family, including GFRα1 to 4, and a remote ligand, GFRAL. RT‐PCR results showed that PNECs expressed *Gfrα1* but not *Gfrα2, Gfrα3*, or *Gfrα4* at the mRNA level (Fig. 1C). Additionally, immunoreactivity against RET was primarily observed on the cell membrane of PNECs, confirming its expression at the protein level (Fig. 1D).

### GDNF expression in an asthmatic mouse lung

**Fig. 2:**
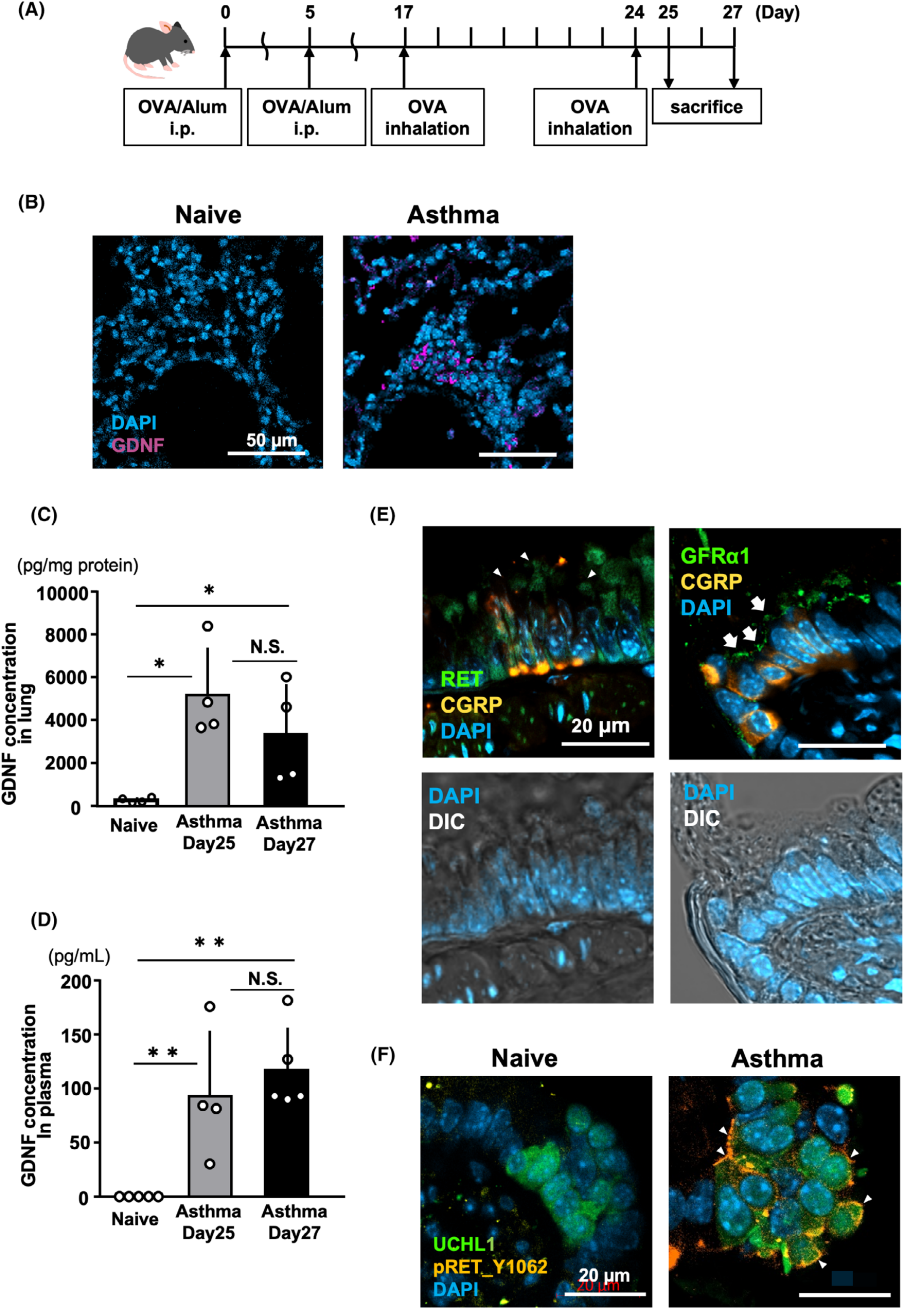
GDNF levels are increased in asthma lungs. (A) Schematic of the OVA‐induced asthma model. (B) Representative images of immunohistochemistry for GDNF‐positive cells in lungs obtained from naïve (left) and day 27 asthma groups (right). Scale bar, 50 μm. Quantitative analyses for GDNF concentrations in lung homogenate (C) and plasma (D). (Lung homogenate; *n* = 4 on naïve, day 25‐ and day 27‐asthma groups, plasma; *n* = 6 on naïve, *n* = 4 on day 25, and *n* = 5 on day 27). The concentration in the naïve group was set to 0 pg mg^−1^, as it was below the detection threshold in (D). Statistical analysis was performed using the Mann–Whitney *U* test. N.S. not significant *p < 0.05, **p < 0.01. Error bars represent mean ± SD. (E) Representative images of immunohistochemistry for RET (left) and GFRα1 (right) in asthmatic lung sections. To identify PNEC, an anti‐CGRP antibody was used. Arrowheads and arrows indicate RET‐ or GFRα1‐positive cells, respectively. Scale bar, 20 μm. (F) Representative images of immunohistochemistry for pRET_Y1062 and UCHL1 in lung sections obtained from naïve (left) or an asthma group (right). Scale bar, 20 μm.

Among the neurotrophic factors, GFRα1, a coreceptor of RET, primarily binds to GDNF. We established an OVA‐induced asthmatic mouse model (Fig. 2A) and analyzed GDNF expression. Under asthmatic conditions, many GDNF‐expressing cells were easily detected in the interstitial area, suggesting that these cells may be infiltrating blood cells. In contrast, GDNF‐expressing cells were rarely visible in naïve lungs (Fig. 2B). Quantitative analysis revealed that GDNF levels in asthmatic lungs increased by over 100‐fold compared with those in naïve lungs (Fig. 2C). GDNF concentrations in the plasma also increased under asthmatic conditions, whereas levels in naïve lungs were below the detection threshold (Fig. 2D). These findings suggest that RET‐mediated GDNF signaling is present only during airway inflammation. We also confirmed PNECs expressed RET and GFRα1 in asthmatic conditions (Fig. 2E). Consistent with these findings, we detected RET phosphorylation at Y1062 in PNECs from asthma mouse models, indicating RET activation (Fig. 2F and Fig. S2). Therefore, we propose that GDNF, produced by infiltrating cells, sequentially binds GFRα1, activates RET, transmits signals within PNECs, and triggers mitogenesis.

### Both macrophages and eosinophils are GDNF‐expressing cells

**Fig. 3:**
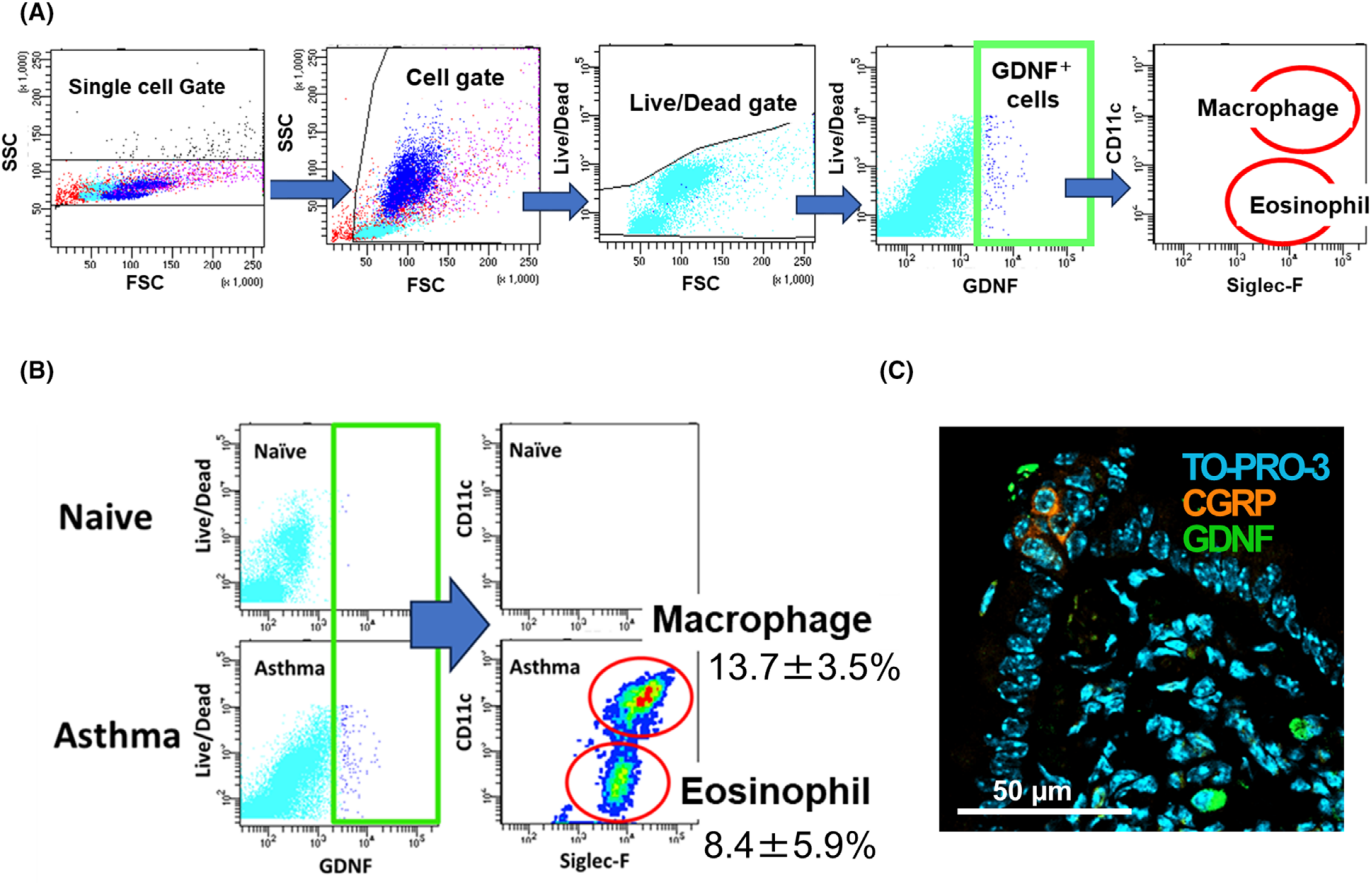
Cells producing GDNF in the asthma lung. (A) Gating strategy for GDNF^+^ cells sorting. Fluorescence‐activated cell sorting plots are representative of the naïve lungs. Eosinophils were identified as Siglec‐F+ and CD11c − cells. Macrophages were identified as Siglec‐F+ and CD11c + cells. (B) Flow cytometry of GDNF^+^ cells in asthma lungs. GDNF+ cells consist of macrophages or eosinophils. (C) Representative images of immunohistochemistry for GDNF (green), CGRP (orange), and TOPRO‐3 (blue) in asthma lung. Scale bar, 50 μm.

Next, we aimed to identify the types of infiltrating cells that expressed GDNF. Fig. 3A shows the overall strategy used for flow cytometry analysis. The results indicated that GDNF‐expressing cells in the asthmatic lungs were primarily macrophages and eosinophils, accounting for 13.7 ± 3.5% and 8.4 ± 5.9%, respectively (Fig. 3B). Immunohistochemistry revealed that GDNF‐expressing cells were often located near the PNEC cluster, within a distance that GDNF released from inflammatory cells could reach in a paracrine manner (Fig. 3C).

### Specific RET inhibitor, pralsetinib, suppressed inflammatory‐inducing PNEC hyperplasia

**Fig. 4:**
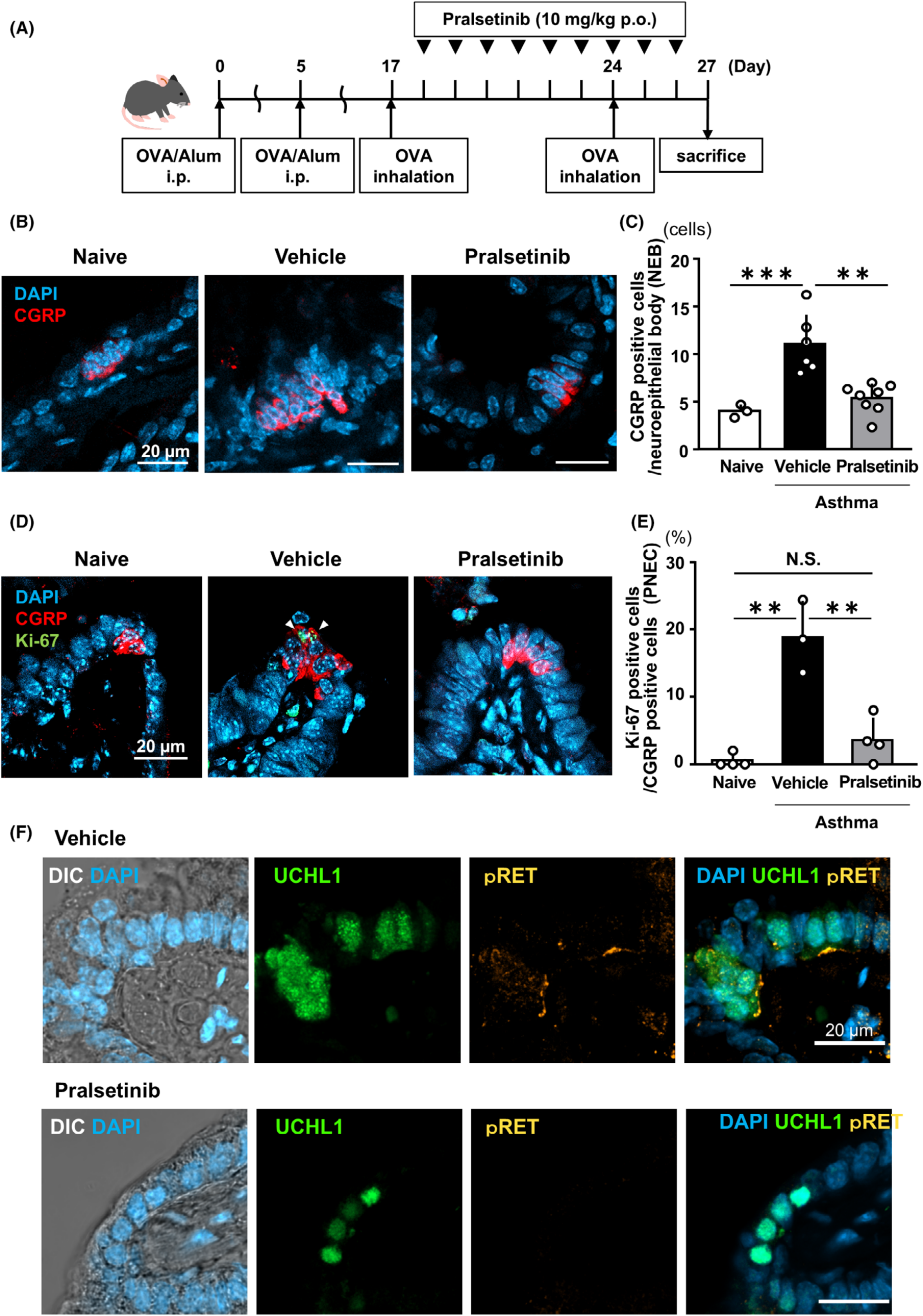
Pralsetinib suppresses PNEC hyperplasia. (A) Schedule of pralsetinib administration in an asthma mouse model. Mice were euthanized on day 27 for analysis. Effect of pralsetinib in OVA‐induced asthma mice (B–F). (B) Representative images of immunohistochemistry for CGRP (red) and DAPI (blue) in lung sections obtained from naïve, vehicle‐treated, or pralsetinib‐treated asthma groups. Scale bar, 20 μm. (*C*) Number of CGRP‐positive PNEC per NEB. Data were analyzed using one‐way ANOVA and Tukey's *post hoc* test (number of mice; *n* = 3 for naïve, *n* = 6 for vehicle‐treated asthma group, and *n* = 8 for pralsetinib‐treated asthma group) **p < 0.01, ***p < 0.001. Error bars represent mean ± SD. (*D*) Representative images of immunohistochemistry for Ki‐67 (green), CGRP (red), and DAPI (blue) in lung sections obtained from naïve, vehicle‐treated, or pralsetinib‐treated asthma lungs. Arrowheads indicate Ki‐67‐positive cells. Scale bar, 20 μm. (E) Number of Ki‐67‐ and CGRP‐positive PNEC per CGRP‐positive PNEC. Data were analyzed using one‐way ANOVA and Tukey's *post hoc* test. (number of mice; *n* = 3 for naïve, *n* = 6 for vehicle‐treated asthma group and *n* = 8 for pralsetinib‐treated asthma group) **p < 0.01. Error bars represent mean ± SD. (F) Representative images of immunohistochemistry for UCHL1 (green), pRET (red), and DAPI (blue) in lung sections obtained from vehicle‐treated (upper) or pralsetinib‐treated (lower) asthma lungs. Scale bar, 20 μm.

Since we proposed that GDNF‐GFRα1‐RET signaling is activated in asthmatic PNECs, we then investigated whether this signaling is crucial for the development of PNEC hyperplasia. We administered a specific RET inhibitor, pralsetinib, to an OVA‐induced asthma mouse model (Fig. 4A). When asthmatic mice were treated with pralsetinib or vehicle for 9 days, PNEC hyperplasia was significantly reduced (Fig 4B,C). This was further supported by a decrease in Ki‐67‐positive cells and in the level of pRET after pralsetinib treatment (Fig 4D,F).

### Treatment with pralsetinib improves asthmatic phenotypes

**Fig. 5:**
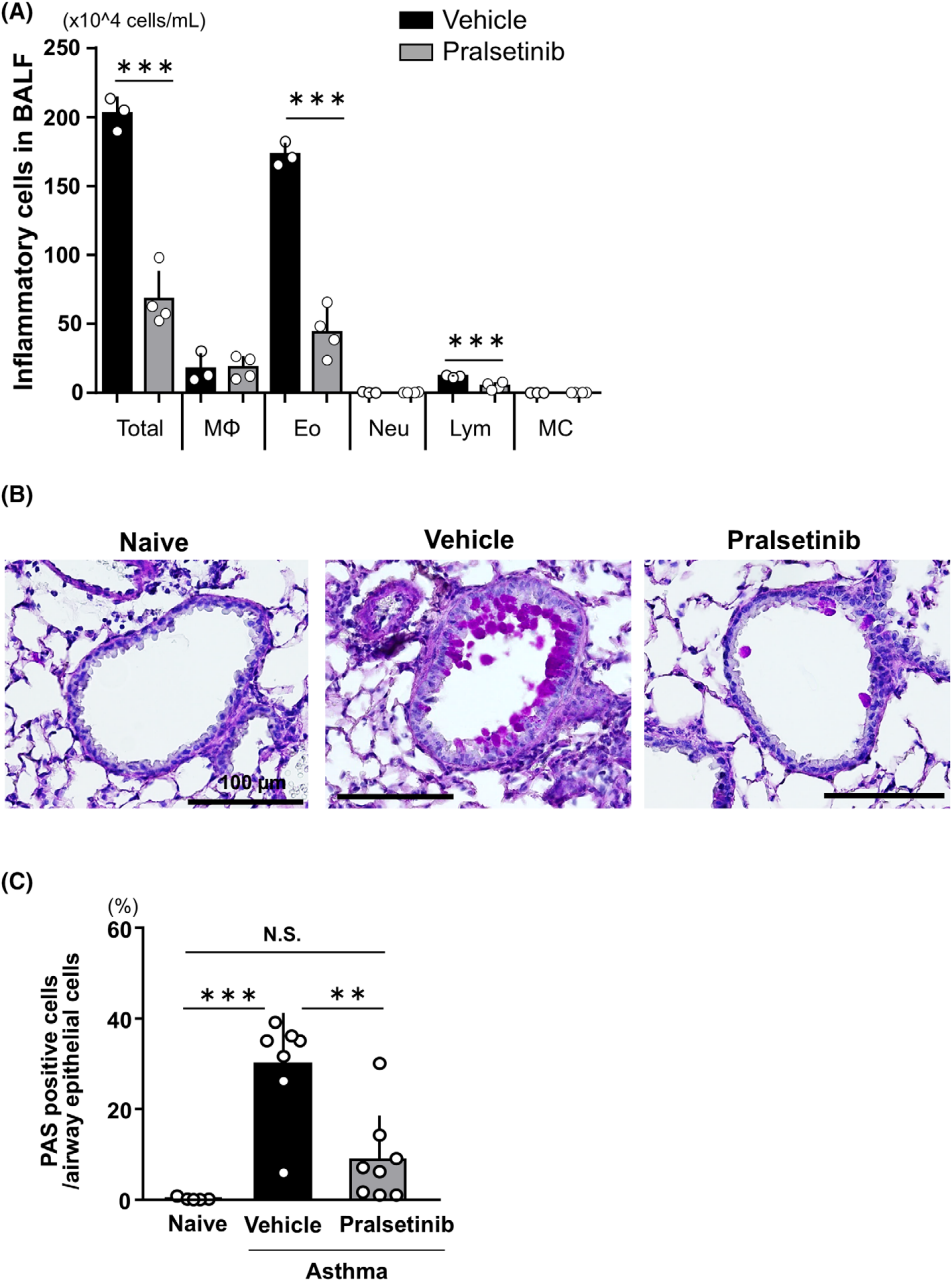
Pralsetinib suppresses the infiltration of inflammatory cells and goblet cell hyperplasia. (A) Total number of cells and constituents in BALF obtained from vehicle‐treated or pralsetinib‐treated asthma group (*n* = 3 for each group). Statistical analysis was performed using Mann–Whitney *U* test. (B) Representative images of PAS staining. Scale bar, 100 μm. (C) Quantitative analysis of PAS‐positive cells (*n* = 6 for the naïve group, *n* = 7 for the vehicle‐treated asthma group, and *n* = 8 for pralsetinib‐treated asthma group). Number of PAS‐positive cells in the airways with an inner diameter of 100–300 μm was counted. Statistical analysis was performed using one‐way ANOVA and Tukey's *post hoc* test. ** *p* < 0.01, *** *p* < 0.001. Error bars represent mean ± SD.

We assessed the anti‐inflammatory effects of pralsetinib on asthmatic response. To examine the cellular infiltration in the airways, BALF was used to measure the total cell count and number of constituent cells in each fraction. The pralsetinib‐treated group showed a reduction in total cell number, as well as in eosinophils and lymphocytes, compared with the vehicle‐treated group (Fig. 5A). Furthermore, lung tissue histology was analyzed using PAS staining, which revealed a significant decrease in the percentage of PAS‐stained cells in the pralsetinib‐treated group (Fig 5B,C). Our results demonstrate that pralsetinib markedly suppresses airway inflammation and goblet cell hyperplasia.

### ILC2 is downregulated in response to pralsetinib

**Fig. 6:**
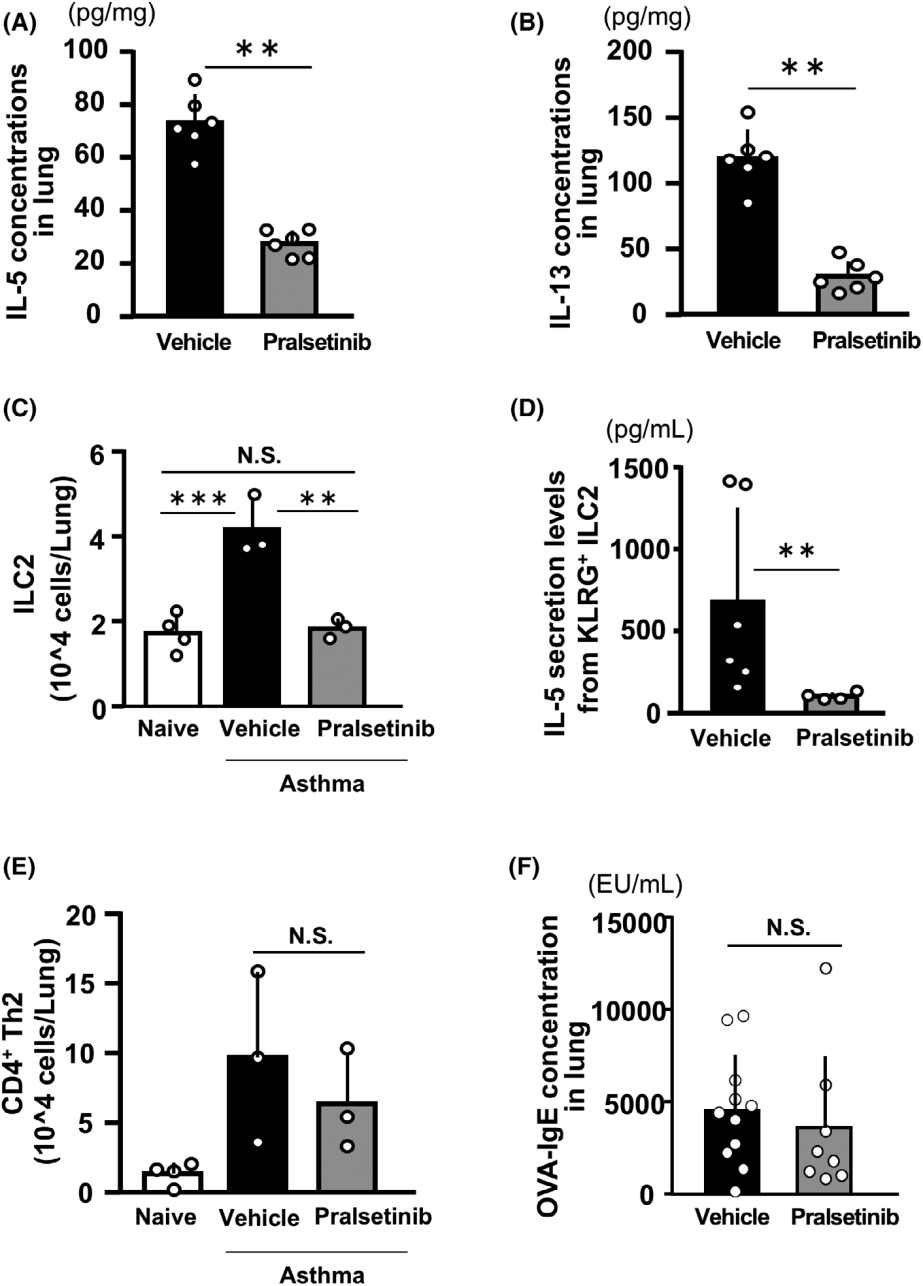
Pralsetinib reduces Th2 cytokine levels and number of ILC2. Concentrations of IL‐5 (A) and IL‐13 (B) in lung homogenates from vehicle‐treated or pralsetinib‐treated asthma groups (*n* = 6 for each group). Mann–Whitney *U* test was used for statistical analysis. **p < 0.01. Error bars represent mean ± SD. Number of KLRG^+^ ILC2 (C) and CD4^+^ Th2 cells (E) sorted by flow cytometry from lungs of naïve, vehicle‐treated, or pralsetinib‐treated groups (*n* = 3 for each group). One‐way ANOVA with Tukey's *post hoc* test was used for statistical analysis. **p < 0.01, ***p < 0.001. Error bars represent mean ± SD. (D) IL‐5 secretion levels from sorted KLRG^+^ ILC2. ILC2 from the vehicle‐ or pralsetinib‐treated asthma groups were stimulated with IL‐33 in culture for 4 days, and IL‐5 levels in the culture supernatants were measured. Mann–Whitney *U* test was used for statistical analysis. **p < 0.01. Error bars represent mean ± SD. (F) OVA‐IgE concentrations in lung homogenates from vehicle‐ or pralsetinib‐treated asthma groups. Student's unpaired *t*‐test was used for statistical analysis (*n* = 12 for the vehicle‐treated group and *n* = 9 for the pralsetinib‐treated group).

We previously reported that CGRP released from PNECs worsened asthmatic phenotypes via ILC2. Therefore, we examined the role of ILC2 in pralsetinib‐treated asthma mice. We measured the concentrations of IL‐5 and IL‐13, which are stored abundantly by ILC2s. The levels of IL‐5 and IL‐13 were significantly lower in the pralsetinib‐treated group than in the vehicle‐treated group (Fig. 6A,B, respectively). When we isolated KLRG^+^ ILC2s and CD4^+^ Th2 cells on the day after the last inhalation and analyzed their cell numbers, we observed a significant decrease in KLRG^+^ ILC2s in the pralsetinib‐treated group compared to the vehicle‐treated group (Fig. 6C). To assess the ability of ILC2s to produce Th2 cytokines, we cultured the isolated KLRG^+^ ILC2s *in vitro* with IL‐33 and measured IL‐5 levels in the resulting culture supernatant. IL‐5 levels were significantly reduced in the pralsetinib‐treated group (Fig. 6D). These data suggest that pralsetinib decreases the number of ILC2s and their capacity to produce Th2 cytokines. Meanwhile, there was almost no difference in the number of CD4^+^ Th2 cells and the OVA‐IgE concentration in lung cells between the pralsetinib‐treated and vehicle‐treated groups (Fig 6E,F). Thus, we propose that suppressing PNEC hyperplasia and CGRP levels with pralsetinib lowers the number and function of ILC2, leading to an improvement in asthma phenotypes.

## Discussion

In our previous study, we showed that a CGRP receptor antagonist reduced typical allergic asthma phenotypes through ILC2 in an OVA‐induced asthma model [14]. In asthmatic lungs, CGRP levels increase along with an abnormal increase in PNECs, known as PNEC hyperplasia. Since it is assumed that CGRP stored abundantly in PNECs triggers ILC2 stimulation, we considered that controlling PNEC hyperplasia would be the most effective way to lower CGRP levels and treat asthma phenotypes. PNEC hyperplasia was first reported in the 1990s [13]. Since then, this phenomenon has often been observed in various lung diseases [12, 26, 27]. However, the precise mechanisms, pathological processes involved, and methods for controlling this condition remain unclear. In this study, we demonstrated that activating RET via GDNF secreted by inflammatory cells is essential for PNEC hyperplasia in an asthma mouse model. Considering the present and previous data, PNEC hyperplasia‐derived CGRP enables ILC2 activation, leading to the release of IL‐5, IL‐13, and other cytokines. IL‐5 stimulates eosinophil infiltration and eosinophil‐derived GDNF induces PNEC hyperplasia through the GDNF receptors, RET and GFRα1. This chain of events seems to indicate a bidirectional neuroimmune relationship centered on the PNEC hyperplasia‐ILC2 axis.

Detailed analysis revealed that the inflammatory cells that produced GDNF were predominantly macrophages and eosinophils (Fig. 3B). Several lines of evidence showed that neuroinflammation induced GDNF expression in infiltrating macrophages [28, 29]. According to a recent study, GDNF levels were significantly higher in the lungs and brains of asthmatic rats, and this increase notably worsened asthma symptoms [30]. Eosinophils also secrete growth factors and cytokines, such as TGF‐β, VEGF, and NGF, in response to immune stimuli [31, 32]. Similarly, our data indicated that eosinophils can produce GDNF in an inflammatory microenvironment (Fig 2B,D).

In experimental animal models, toxic naphthalene injection results in rapid PNEC hyperplasia, which is thought to be triggered by injury to the acute airway epithelium, particularly club cells [33, 34]. However, our asthma model did not show significant cell injury or loss of the bronchiole epithelium, unlike the naphthalene‐induced models. PNEC hyperplasia is also associated with chronic hypoxia, such as living at high altitudes [35] or the genetic deletion of prolyl hydroxylase domain enzymes 1–3, which catalyze the hydroxylation of hypoxia‐inducible factor 1 in response to hypoxia [36]. Additionally, the loss of NOTCH signaling molecules, including hairy and enhancer of split 1 (HES‐1) or some Notch ligands, induces PNEC hyperplasia [37, 38, 39]. Currently, it is difficult to explain the mechanisms underlying PNEC hyperplasia using a single concept, as they may vary depending on the type of trigger, situation, or duration (temporary or ongoing). Further research is needed to clarify whether GDNF‐RET signaling alone is sufficient to cause PNEC hyperplasia outside of the asthma model. Notably, the carotid body, which exhibits hypoxia‐induced hyperplasia, expresses GDNF, RET, and GDNF family receptor α, contributes to its mitogenesis, at least *in vitro* [40, 41, 42].

In this study, we demonstrated the therapeutic effects of pralsetinib, a RET‐specific inhibitor, in an OVA‐induced asthma model. Pralsetinib suppressed PNEC hyperplasia and the typical features of allergic asthma, including inflammatory cell infiltration and goblet cell hyperplasia (Fig 5A,C). Pralsetinib has been approved for the treatment of advanced or metastatic RET‐mutant medullary thyroid cancer and metastatic RET fusion‐positive non‐small‐cell lung cancer by the Food and Drug Administration in the United States. Although the efficacy and specificity of pralsetinib have been demonstrated in clinical practice, we cannot rule out the possibility that its effects may extend beyond the target, resulting in off‐target effects. Additionally, we believe that one of the major targets of pralsetinib in the lungs is PNEC, as indicated by RT‐qPCR and IHC for RET (Fig. 1B,D, respectively). A recent study reported that adipose‐resident ILC2s express RET [43]. To analyze whether lung‐derived ILC2s express RET, we performed immunocytochemistry against RET on sorted ILC2s. We did not detect RET immunoreactivity, suggesting low or no RET expression in lung‐derived ILC2 (Fig. S3). We also observed a decrease in goblet cells in pralsetinib‐treated asthma mice compared to vehicle‐treated mice (Fig 5B,C). Through the neurotransmitter GABA, PNECs are reported to act to induce goblet‐cell hyperplasia, and the absence of PNECs leads to a reduction in GABA along with key neuropeptides [11]. Therefore, we suggest that the decrease in goblet cells results from the reduction of GABA, which is caused by the downregulation of PNECs due to pralsetinib. In other diseases involving PNEC hyperplasia, such as DIPNECH and NEHI, no effective treatment has been established yet. Therefore, the treatment proposed in this study might provide a clue, although further verification is needed.

Our data highlight the significance of GDNF‐RET signaling during the acute phase of asthma development. Prakash *et al*. also noted that GDNF enhanced mixed allergen‐induced airway hyperreactivity and tissue remodeling in the later chronic phase of another asthma model (Fig. S4) [44]. Considering both datasets, GDNF‐RET signaling may be crucial in both the acute and chronic phases of asthma.

In conclusion, we clarified that the bidirectional neuroimmune interaction between PNECs and immune cells, such as ILC2, eosinophils, and macrophages, may play a pivotal role in the development of asthma. Targeting this interaction therapeutically could improve asthma phenotypes.

## Author contributions

TK and TT conceived and designed the project. TK and EI acquired the data. TK, EI, SO, and TT analyzed and interpreted the data. TK and TT wrote the paper.

## Conflict of interest

The authors declare no conflict of interest.

## Supporting information


**Fig. S1.** The gating strategy for isolating ILC2 and Th2.
**Fig. S2.** Immunohistochemistry for RET and pRET_Y1062 in PNECs.
**Fig. S3.** Immunohistochemistry for RET in airway smooth muscle cells.
**Fig. S4.** Immunocytochemistry for RET in ILC2.
**Table S1.** Antibodies used for flow cytometry–based cell isolation.
**Table S2.** Primers used in RT‐PCR.
**Table S3.** Primary antibodies used in immunohistochemistry.

## Data Availability

The data underlying this article are available in the Dryad Digital Repository at DOI: https://doi.org/10.5061/dryad.cfxpnvxmm
