# Supplementary material for: GDNF‐RET signaling drives pulmonary neuroendocrine cell hyperplasia and allergic airway inflammation
Source: FEBS Lett. 2026 Apr 12;600(8):1185–98. doi: 10.1002/1873-3468.70341 (PMC13113197; doi:10.1002/1873-3468.70341)
Supplement: Supplementary file 1 — Fig. S1. The gating strategy for isolating ILC2 and Th2. Fig. S2. Immunohistochemistry for RET and pRET_Y1062 in PNECs. Fig. S3. Immunohistochemistry for RET in airway smooth muscle cells. Fig. S4. Immunocytochemistry for RET in ILC2. Table S1. Antibodies used for flow cytometry–based cell isolation. Table S2. Primers used in RT‐PCR. Table S3. Primary antibodies used in immunohistochemistry. [file FEB2-600-1185-s001.pptx]

## Slide 1
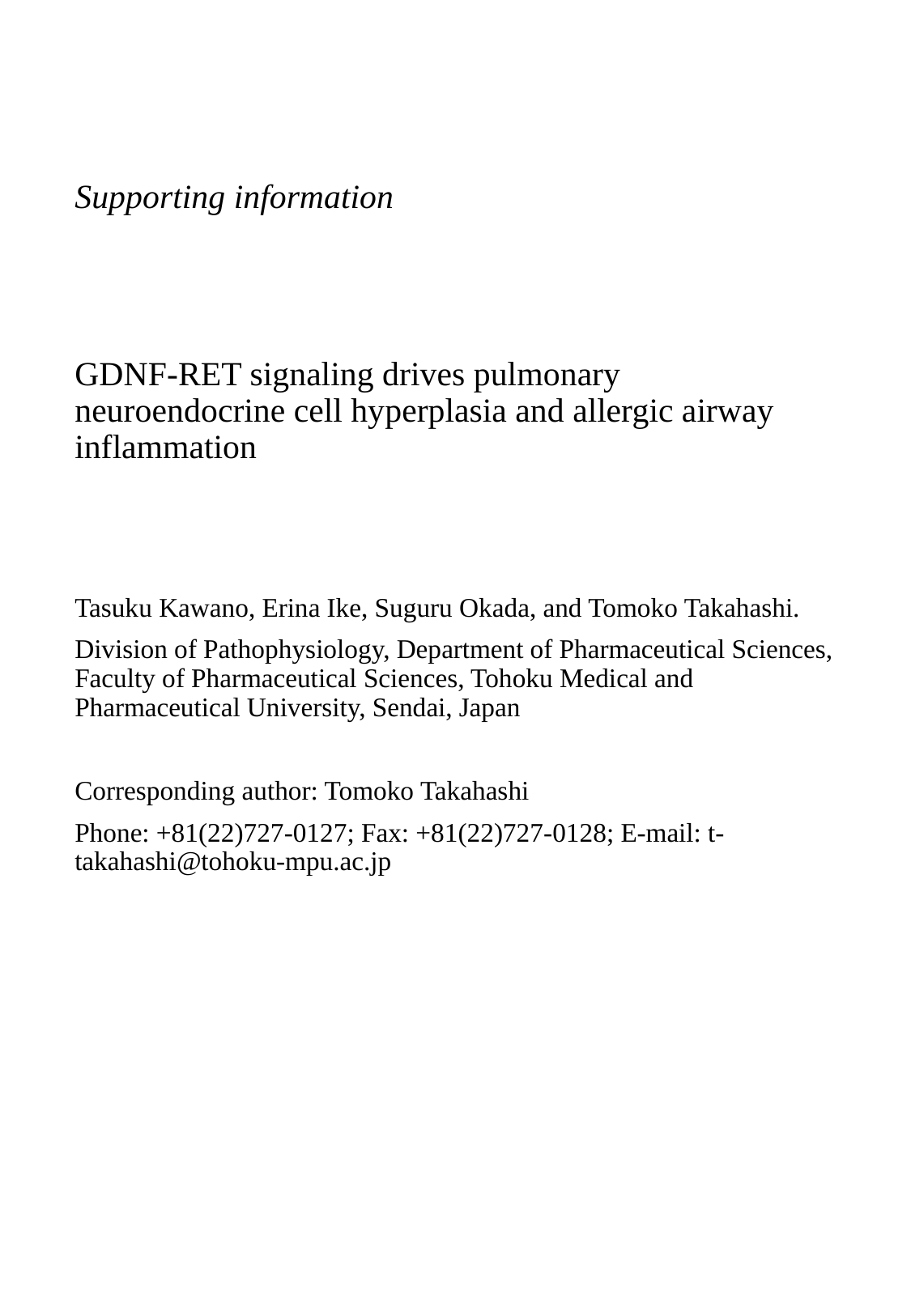

# Supporting information
GDNF-RET signaling drives pulmonary neuroendocrine cell hyperplasia and allergic airway inflammation
Tasuku Kawano, Erina Ike, Suguru Okada, and Tomoko Takahashi.
Division of Pathophysiology, Department of Pharmaceutical Sciences, Faculty of Pharmaceutical Sciences, Tohoku Medical and Pharmaceutical University, Sendai, Japan
Corresponding author: Tomoko Takahashi
Phone: +81(22)727-0127; Fax: +81(22)727-0128; E-mail: t-takahashi@tohoku-mpu.ac.jp

## Slide 2
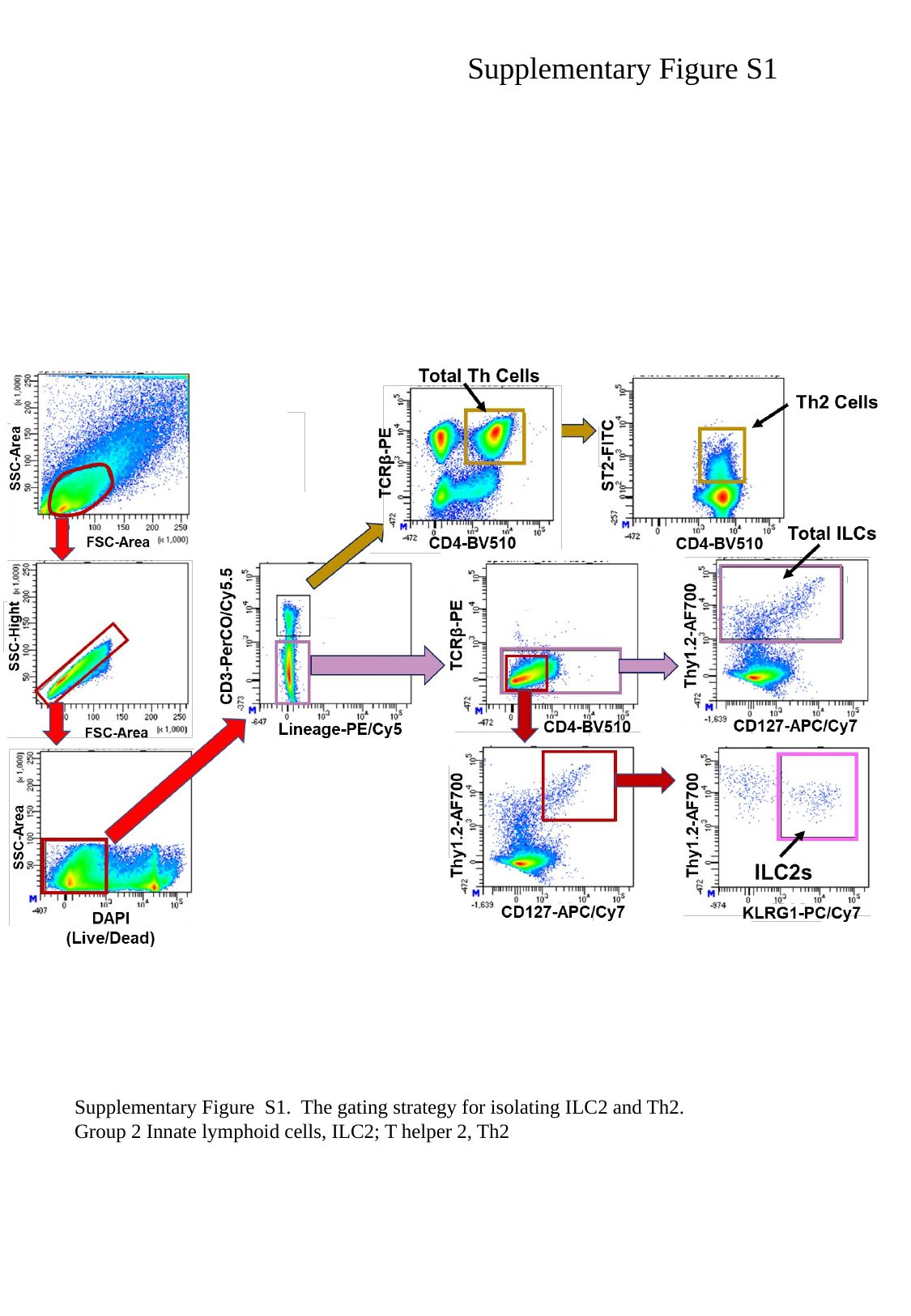

Supplementary Figure S1
Supplementary Figure S1. The gating strategy for isolating ILC2 and Th2.
Group 2 Innate lymphoid cells, ILC2; T helper 2, Th2

## Slide 3
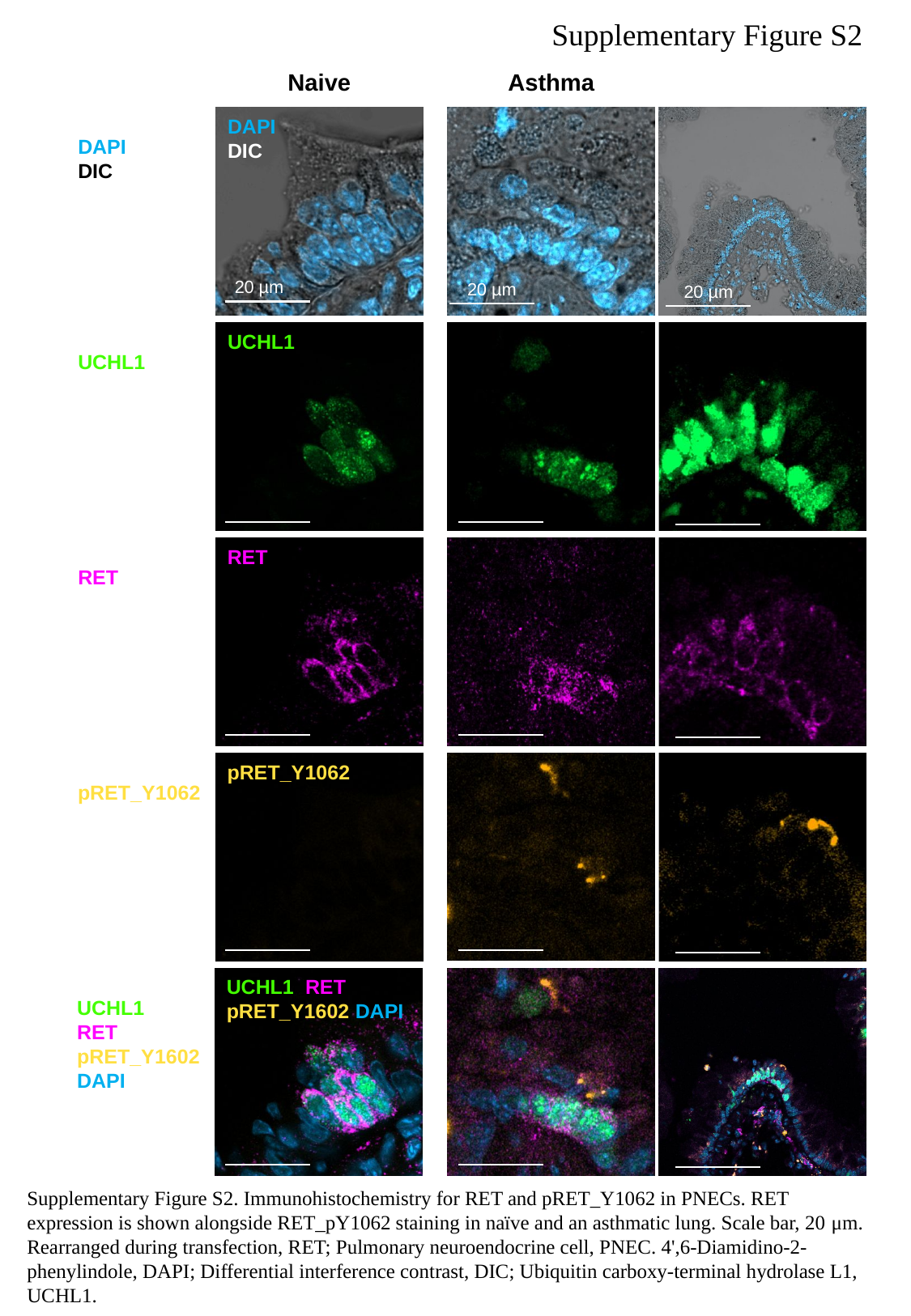

Supplementary Figure S2
Naive
Asthma
DAPI
DIC
DAPI
DIC
UCHL1
RET
pRET_Y1062
UCHL1
RET
pRET_Y1602
DAPI
20 µm
20 µm
20 µm
UCHL1
RET
pRET_Y1062
UCHL1 RET pRET_Y1602 DAPI
Supplementary Figure S2. Immunohistochemistry for RET and pRET_Y1062 in PNECs. RET expression is shown alongside RET_pY1062 staining in naïve and an asthmatic lung. Scale bar, 20 μm. Rearranged during transfection, RET; Pulmonary neuroendocrine cell, PNEC. 4',6-Diamidino-2-phenylindole, DAPI; Differential interference contrast, DIC; Ubiquitin carboxy-terminal hydrolase L1, UCHL1.

## Slide 4
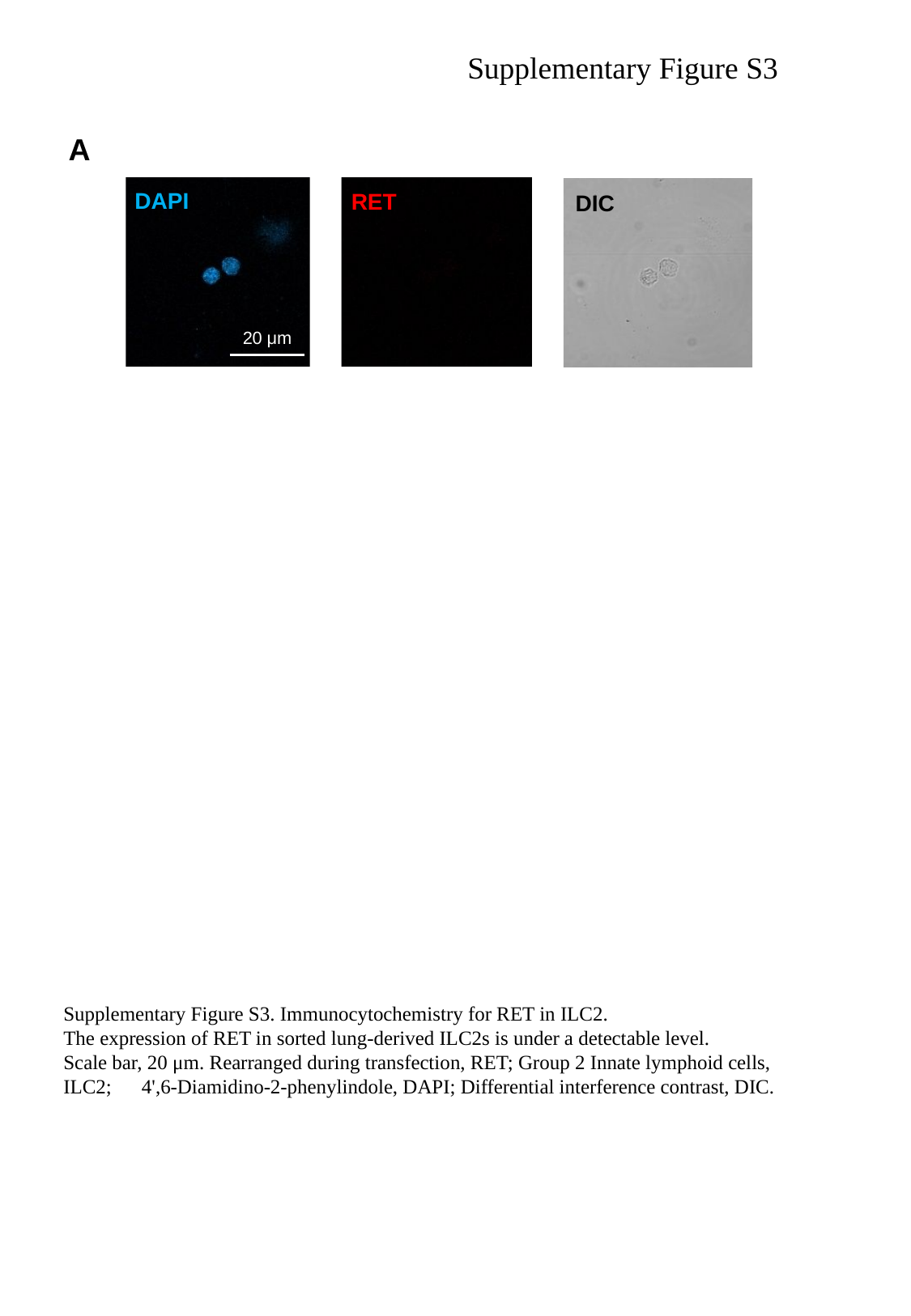

Supplementary Figure S3
A
DAPI
20 μm
RET
DIC
Supplementary Figure S3. Immunocytochemistry for RET in ILC2.
The expression of RET in sorted lung-derived ILC2s is under a detectable level.
Scale bar, 20 μm. Rearranged during transfection, RET; Group 2 Innate lymphoid cells, ILC2;　4',6-Diamidino-2-phenylindole, DAPI; Differential interference contrast, DIC.

## Slide 5
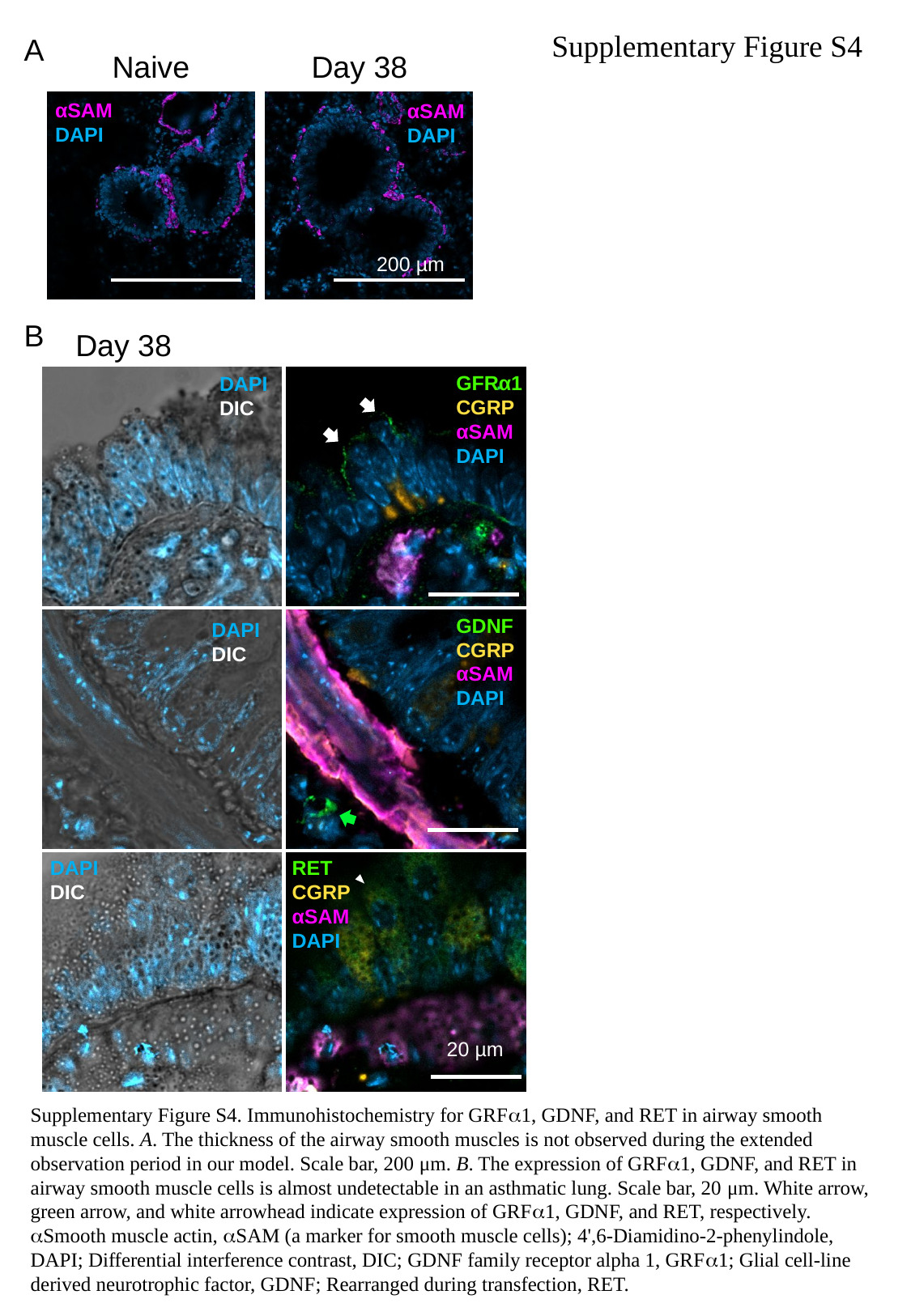

A
Supplementary Figure S4
Naive
Day 38
αSAM
DAPI
αSAM
DAPI
200 µm
B
Day 38
GFRα1
CGRP
αSAM
DAPI
DAPI
DIC
GDNF
CGRP
αSAM
DAPI
DAPI
DIC
RET
CGRP
αSAM
DAPI
DAPI
DIC
20 µm
Supplementary Figure S4. Immunohistochemistry for GRFa1, GDNF, and RET in airway smooth muscle cells. A. The thickness of the airway smooth muscles is not observed during the extended observation period in our model. Scale bar, 200 μm. B. The expression of GRFa1, GDNF, and RET in airway smooth muscle cells is almost undetectable in an asthmatic lung. Scale bar, 20 μm. White arrow, green arrow, and white arrowhead indicate expression of GRFa1, GDNF, and RET, respectively. aSmooth muscle actin, aSAM (a marker for smooth muscle cells); 4',6-Diamidino-2-phenylindole, DAPI; Differential interference contrast, DIC; GDNF family receptor alpha 1, GRFa1; Glial cell-line derived neurotrophic factor, GDNF; Rearranged during transfection, RET.

## Slide 6
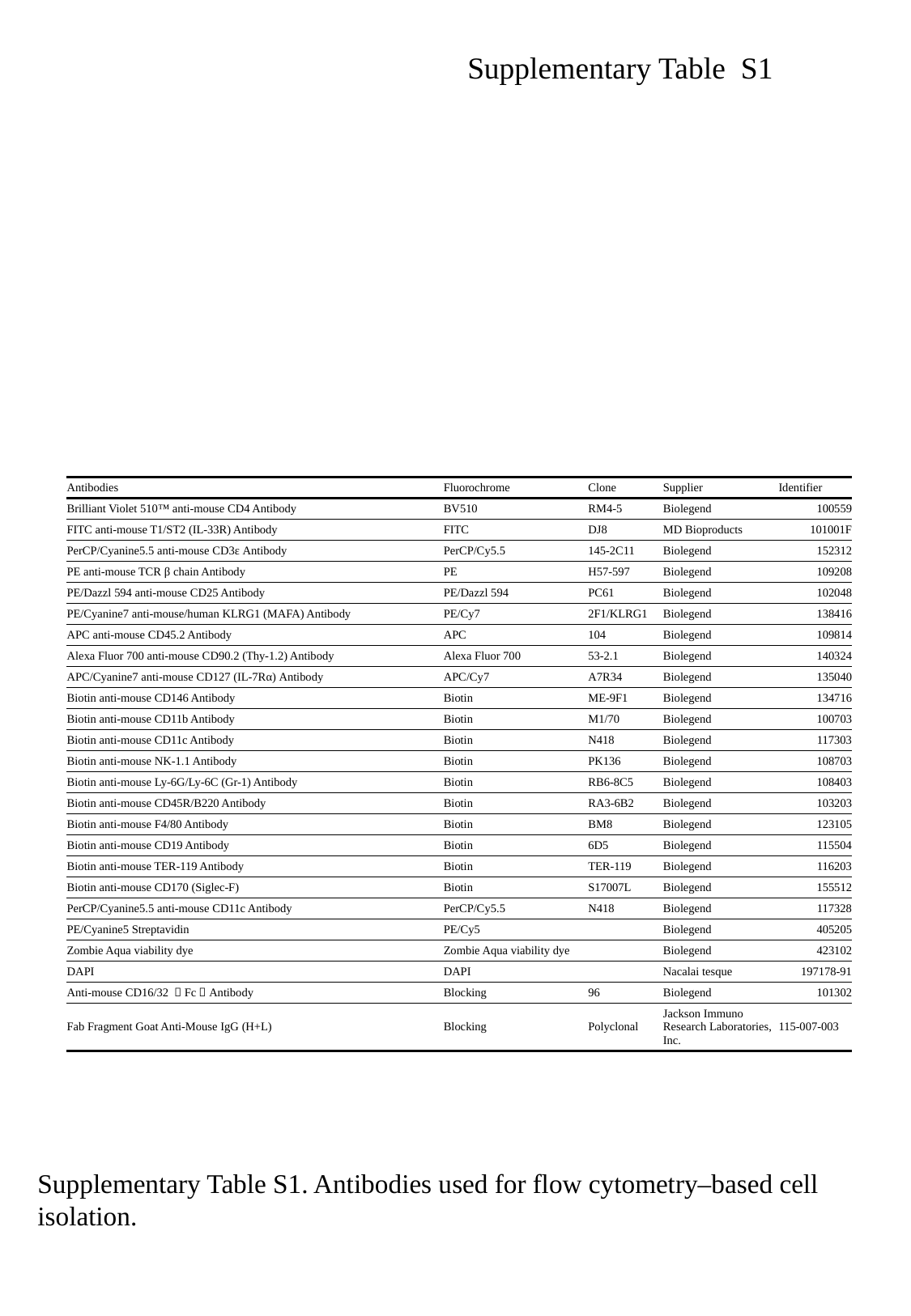

Supplementary Table S1
| Antibodies | Fluorochrome | Clone | Supplier | Identifier |
| --- | --- | --- | --- | --- |
| Brilliant Violet 510™ anti-mouse CD4 Antibody | BV510 | RM4-5 | Biolegend | 100559 |
| FITC anti-mouse T1/ST2 (IL-33R) Antibody | FITC | DJ8 | MD Bioproducts | 101001F |
| PerCP/Cyanine5.5 anti-mouse CD3ε Antibody | PerCP/Cy5.5 | 145-2C11 | Biolegend | 152312 |
| PE anti-mouse TCR β chain Antibody | PE | H57-597 | Biolegend | 109208 |
| PE/Dazzl 594 anti-mouse CD25 Antibody | PE/Dazzl 594 | PC61 | Biolegend | 102048 |
| PE/Cyanine7 anti-mouse/human KLRG1 (MAFA) Antibody | PE/Cy7 | 2F1/KLRG1 | Biolegend | 138416 |
| APC anti-mouse CD45.2 Antibody | APC | 104 | Biolegend | 109814 |
| Alexa Fluor 700 anti-mouse CD90.2 (Thy-1.2) Antibody | Alexa Fluor 700 | 53-2.1 | Biolegend | 140324 |
| APC/Cyanine7 anti-mouse CD127 (IL-7Rα) Antibody | APC/Cy7 | A7R34 | Biolegend | 135040 |
| Biotin anti-mouse CD146 Antibody | Biotin | ME-9F1 | Biolegend | 134716 |
| Biotin anti-mouse CD11b Antibody | Biotin | M1/70 | Biolegend | 100703 |
| Biotin anti-mouse CD11c Antibody | Biotin | N418 | Biolegend | 117303 |
| Biotin anti-mouse NK-1.1 Antibody | Biotin | PK136 | Biolegend | 108703 |
| Biotin anti-mouse Ly-6G/Ly-6C (Gr-1) Antibody | Biotin | RB6-8C5 | Biolegend | 108403 |
| Biotin anti-mouse CD45R/B220 Antibody | Biotin | RA3-6B2 | Biolegend | 103203 |
| Biotin anti-mouse F4/80 Antibody | Biotin | BM8 | Biolegend | 123105 |
| Biotin anti-mouse CD19 Antibody | Biotin | 6D5 | Biolegend | 115504 |
| Biotin anti-mouse TER-119 Antibody | Biotin | TER-119 | Biolegend | 116203 |
| Biotin anti-mouse CD170 (Siglec-F) | Biotin | S17007L | Biolegend | 155512 |
| PerCP/Cyanine5.5 anti-mouse CD11c Antibody | PerCP/Cy5.5 | N418 | Biolegend | 117328 |
| PE/Cyanine5 Streptavidin | PE/Cy5 | | Biolegend | 405205 |
| Zombie Aqua viability dye | Zombie Aqua viability dye | | Biolegend | 423102 |
| DAPI | DAPI | | Nacalai tesque | 197178-91 |
| Anti-mouse CD16/32 （Fc）Antibody | Blocking | 96 | Biolegend | 101302 |
| Fab Fragment Goat Anti-Mouse IgG (H+L) | Blocking | Polyclonal | Jackson Immuno Research Laboratories, Inc. | 115-007-003 |
Supplementary Table S1. Antibodies used for flow cytometry–based cell isolation.

## Slide 7
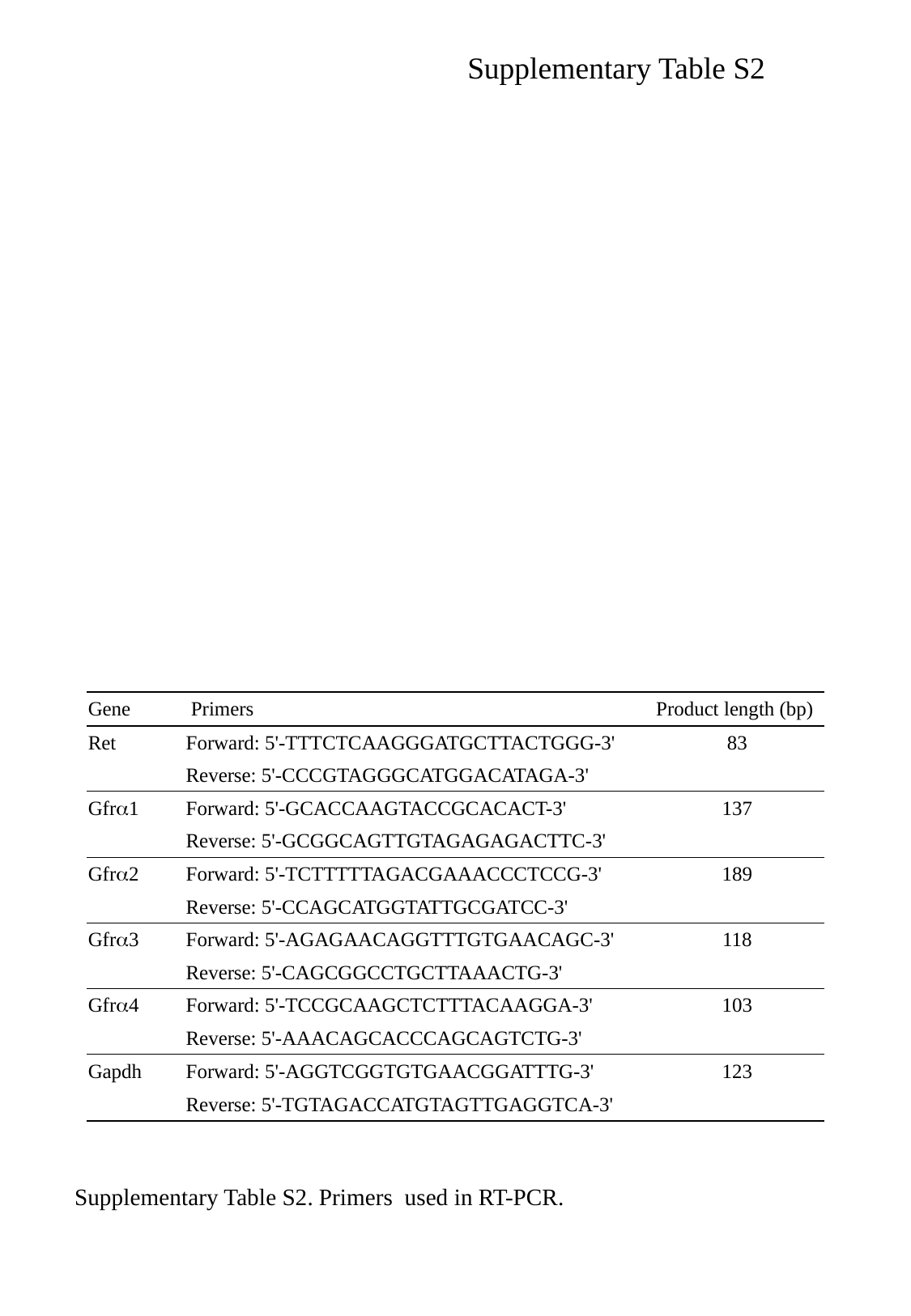

Supplementary Table S2
| | | |
| --- | --- | --- |
| Gene | Primers | Product length (bp) |
| Ret | Forward: 5'-TTTCTCAAGGGATGCTTACTGGG-3' | 83 |
| | Reverse: 5'-CCCGTAGGGCATGGACATAGA-3' | |
| Gfra1 | Forward: 5'-GCACCAAGTACCGCACACT-3' | 137 |
| | Reverse: 5'-GCGGCAGTTGTAGAGAGACTTC-3' | |
| Gfra2 | Forward: 5'-TCTTTTTAGACGAAACCCTCCG-3' | 189 |
| | Reverse: 5'-CCAGCATGGTATTGCGATCC-3' | |
| Gfra3 | Forward: 5'-AGAGAACAGGTTTGTGAACAGC-3' | 118 |
| | Reverse: 5'-CAGCGGCCTGCTTAAACTG-3' | |
| Gfra4 | Forward: 5'-TCCGCAAGCTCTTTACAAGGA-3' | 103 |
| | Reverse: 5'-AAACAGCACCCAGCAGTCTG-3' | |
| Gapdh | Forward: 5'-AGGTCGGTGTGAACGGATTTG-3' | 123 |
| | Reverse: 5'-TGTAGACCATGTAGTTGAGGTCA-3' | |
Supplementary Table S2. Primers used in RT-PCR.

## Slide 8
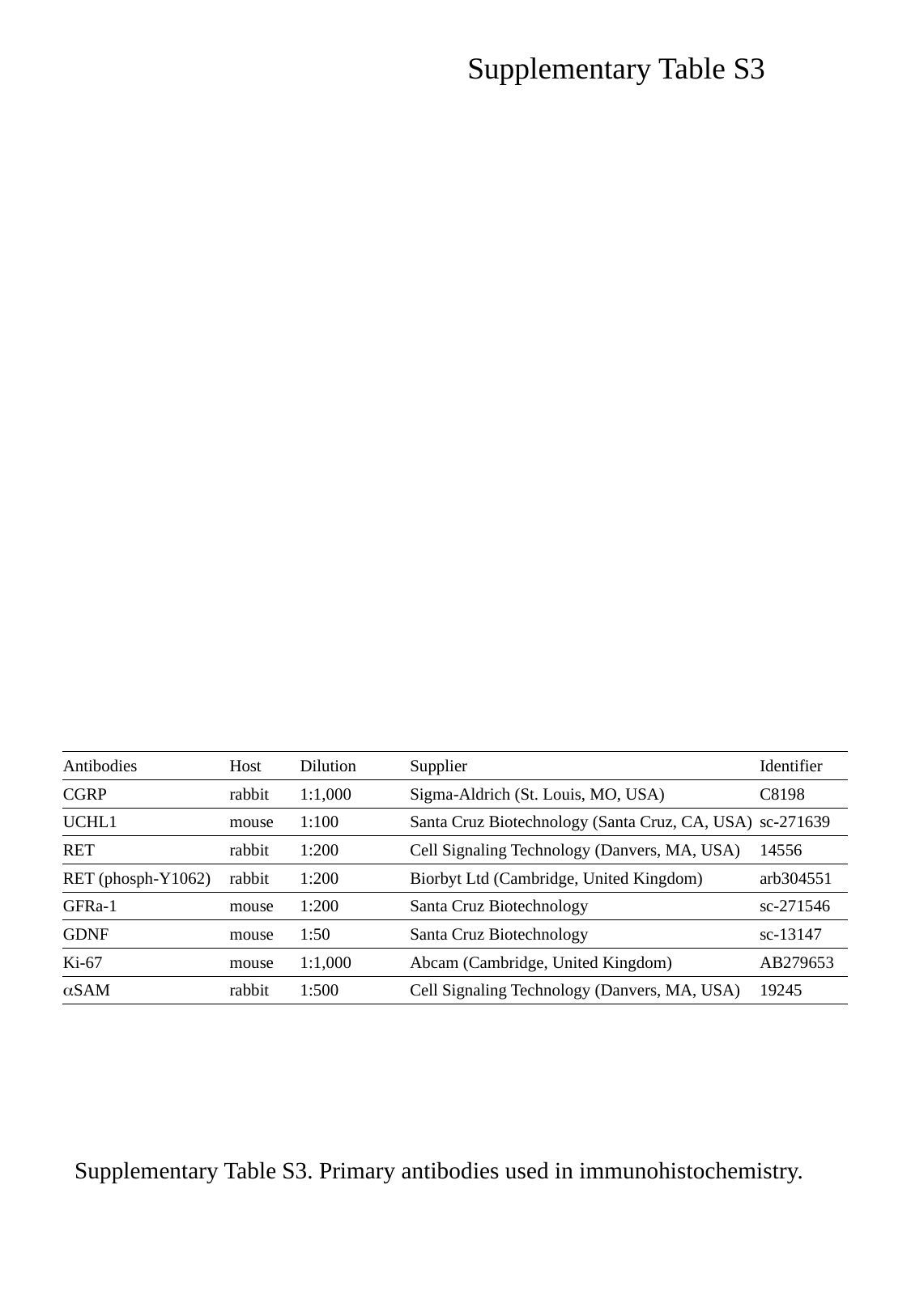

Supplementary Table S3
| Antibodies | Host | Dilution | Supplier | Identifier |
| --- | --- | --- | --- | --- |
| CGRP | rabbit | 1:1,000 | Sigma-Aldrich (St. Louis, MO, USA) | C8198 |
| UCHL1 | mouse | 1:100 | Santa Cruz Biotechnology (Santa Cruz, CA, USA) | sc-271639 |
| RET | rabbit | 1:200 | Cell Signaling Technology (Danvers, MA, USA) | 14556 |
| RET (phosph-Y1062) | rabbit | 1:200 | Biorbyt Ltd (Cambridge, United Kingdom) | arb304551 |
| GFRa-1 | mouse | 1:200 | Santa Cruz Biotechnology | sc-271546 |
| GDNF | mouse | 1:50 | Santa Cruz Biotechnology | sc-13147 |
| Ki-67 | mouse | 1:1,000 | Abcam (Cambridge, United Kingdom) | AB279653 |
| aSAM | rabbit | 1:500 | Cell Signaling Technology (Danvers, MA, USA) | 19245 |
Supplementary Table S3. Primary antibodies used in immunohistochemistry.
